# The impact of herpes zoster and post-herpetic neuralgia on quality-of-life

**DOI:** 10.1186/1741-7015-8-37

**Published:** 2010-06-21

**Authors:** Robert W Johnson, Didier Bouhassira, George Kassianos, Alain Leplège, Kenneth E Schmader, Thomas Weinke

**Affiliations:** 1 9 Ridgeway Road, Long Ashton, Bristol BS41 9EX, UK; 2 INSERM U987, Hôpital Ambroise Paré, APHP, F-92100 Boulogne-Billancourt, France; 3 61 Plough Lane, Wokingham, Berkshire, RG40 1RQ, UK; 4 Université Paris Diderot, Département Histoire et Philosophie des sciences, Bâtiment les Grands Moulins - Case 7019, 75205 Paris cedex 13, France; 5 Durham Veterans Administration, Medical Center (VAMC), Division of Geriatrics, DUMC 3469, Durham, NC 27710, USA; 6 Klinikum Ernst von Bergmann, Gastroenterology and Infectious Diseases, Charlottenstr. 72, 14467 Potsdam, Germany

## Abstract

**Background:**

The potentially serious nature of herpes zoster (HZ) and the long-term complication post-herpetic neuralgia (PHN) are often underestimated. One in four people will contract herpes zoster in their lifetime, with this risk rising markedly after the age of 50 years, and affecting one in two in elderly individuals. Pain is the predominant symptom in all phases of HZ disease, being reported by up to 90% of patients. In the acute phase, pain is usually moderate or severe, with patients ranking HZ pain as more intense than post-surgical or labour pains. Up to 20% of patients with HZ develop PHN, which is moderate-to-severe chronic pain persisting for months or years after the acute phase. We review the available data on the effect of HZ and PHN on patients' quality-of-life.

**Discussion:**

Findings show that HZ, and particularly PHN, have a major impact on patients' lives across all four health domains - physical, psychological, functional and social. There is a clear correlation between increasing severity of pain and greater interference with daily activities. Non-pain complications such as HZ ophthalmicus can increase the risk of permanent physical impairment. Some elderly individuals may experience a permanent loss of independence after an acute episode of HZ. Current challenges in the management of HZ and PHN are highlighted, including the difficulty in administering antiviral agents before pain becomes established and the limited efficacy of pain treatments in many patients. We discuss the clinical rationale for the HZ vaccine and evidence demonstrating that the vaccine reduces the burden of the disease. The Shingles Prevention Study, conducted among >38,000 people aged ≥60 years old, showed that the HZ vaccine significantly reduces the burden of illness and the incidence of both HZ and PHN. In the entire study population, zoster vaccination reduced the severity of interference of HZ and PHN with activities of daily living by two-thirds, as measured by two questionnaires specific to HZ.

**Summary:**

A vaccination scheme may positively impact the incidence and course of HZ disease, thereby improving patients' quality-of-life.

## Background

Herpes zoster (HZ) is a common, painful and debilitating condition caused by a reactivation of the varicella-zoster virus (VZV) from a latent infection of sensory ganglia [1-3]. The disease course can be divided into four phases: prodrome, acute, subacute and chronic [4]. The prodrome occurs 1-5 days before the onset of HZ rash in 70%-80% of cases [5]. Symptoms are non-specific and range from itching to an intense burning sensation [6]. General constitutional symptoms (for example, fever, malaise, headaches) may also occur [5]. The acute phase of HZ disease is characterised by a vesicular skin rash in the affected dermatome which is usually accompanied by acute pain [5,7]. Acute HZ is usually defined as occurring up to 30 days after rash onset [8]. In patients who subsequently develop chronic disease, there is a subacute phase (30-90 days after rash onset) [9].

Post-herpetic neuralgia (PHN) is the most common complication of HZ. A standard definition for PHN is lacking, but it is often defined as pain that persists for ≥90 days after the onset of HZ rash [8]. The chronic pain of PHN is debilitating and can persist for months or years after the acute disease phase [10,11]. Studies vary in the reporting of the duration of persistent pain [12]. In one study of patients aged ≥65 years, the mean duration of pain was 3.3 years, and ranged from 3 months to more than 10 years [13].

The lifetime risk of contracting HZ is one in four, but this risk increases markedly after 50 years of age due to an age-related decline in VZV-specific cell-mediated immunity [6,14,15]. PHN incidence also increases rapidly in individuals after the age of 60 years [16,17]. Of patients with HZ who are ≥50 years old, as many as 10%-20% will develop PHN [11].

HZ is incorrectly perceived by many clinicians to be a mild and readily treatable disease. In fact, treatment options remain inadequate -- particularly for PHN -- and the disease can have devastating effects on patients' quality-of-life (QoL) in the acute and chronic phases [13,18,19]. These effects are widespread, affecting patients' physical and psychological health, as well as their ability to continue normal daily and social activities. Non-pain complications (for example, ophthalmic, neurological) are also a problem in patients with HZ [20].

This contribution discusses the available data on the impact of HZ and PHN on the QoL and functional status of patients and highlights the current challenges in their management. An increased awareness of the substantial burden of HZ and PHN on QoL is essential and may lead to an improvement in prevention and management strategies.

## Discussion

The World Health Organization defines health as 'a state of complete physical, mental and social wellbeing, and not merely the absence of disease or infirmity' [21]. The term QoL, describes the overall sense of wellbeing that a person is experiencing, and how well that person can live a life that is normal for them [22]. Elements of QoL include basic activities (for example, managing to bathe, dress and eat), complex activities (for example, shopping and carrying out household chores), emotional wellbeing (for example, level of fear, anxiety, distress and ability to concentrate) and enjoying relationships with family, friends and social groups.

Clinical studies should assess all aspects of the life and health of a patient in order to establish how a disease or treatment affects their QoL [23]. Health-related QoL instruments measure four key health domains -- physical, psychological, social and functional (the latter includes the basic activities of daily living; ADL [18,23,24].

In patients with HZ and PHN, the QoL analyses rely on subjective self-reporting using generic, standardized and validated health-status questionnaires such as EuroQoL and Short form (SF)-12 [19]. These generic tools are used to measure the impact of a disease on QoL consistently across the four domains of health in all types of patients and general populations [25]. The McGill Pain Questionnaire Present Pain Intensity (a generic tool for pain assessment) is also frequently used because pain is part of health status in the context of diseases such as HZ and PHN [19].

Two questionnaires have been specifically developed to assess how the pain and discomfort associated with HZ and PHN diminish functional capacity: the Zoster Brief Pain Inventory (ZBPI) [19,26] and the Zoster Impact Questionnaire (ZIQ) [19]. The ZBPI measures the impact of the pain and discomfort caused by HZ or PHN on the ability to carry out seven general activities: work, sleep, walking, life enjoyment, mood, relations with others and general activity [19,26]. This tool, therefore, determines the effect of HZ and PHN on QoL across all four domains of health. The ZIQ assesses how HZ pain and PHN affect the functional aspects of life (bathing, grooming, dressing, concentration, preparing meals, eating meals, housework, leisure activities, leaving the house, shopping, travelling). The ZIQ is more focused on daily activities than the ZBPI [19]. The ZBPI has been evaluated against other validated pain questionnaires, but the ZIQ has not [19]. Both of these tools are recognized as sensitive and reliable measures of the impact of HZ and PHN on patients' daily lives [19,27].

### Impact of HZ-related pain on QoL and ADL

Pain is usually the predominant symptom in all phases of HZ disease, being reported by up to 90% of patients [28]. The intensity of pain in patients with HZ and PHN is assessed using subjective self-reporting. Patients are asked to rate their level of HZ/PHN pain (for on average or at its worst) on a numerical rating scale from 0 (no pain) to 10 (pain as bad as you can imagine).

#### Features of HZ pain

Most patients with acute HZ experience moderate or severe dermatomal pain in the skin innervated by the afflicted ganglion [11]. Acute-phase pain is due to the damage to neuronal tissues as a result of viral replication and immune responses [1,29]. Patients rank pain during the acute phase as more intense than post-surgical or labour pain [30].

Pain in the acute phase can be constant or intermittent and may show varied symptoms, such as burning or stabbing sensations, itching, tingling or numbness, angina-like aching or squeezing sensations and a deep aching or 'pulled muscle' sensation [10]. Many patients present with tactile allodynia (pain from a stimulus that is not normally painful) such as the touch of clothing or a light breeze across the skin [10]. Allodynia can be very disabling and may predict a higher risk of developing PHN [9]. Other PHN predictive factors include the presence of prodromal pain, severity of rash, severity of pain and ophthalmic localization [31-35].

#### Impact of HZ pain on Qol

**Figure 1:**
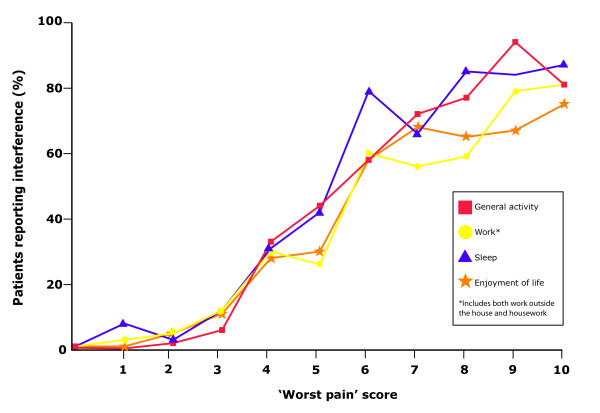
**Association between 'worst pain' score and some interference with individual activities of daily living **[36]. Fifty patients with HZ were asked to rate their level of pain on a scale from 0 (no pain) to 10 (pain as bad as you can imagine). Interference of pain with seven activities of daily living was measured by the Wisconsin Brief Pain Inventory (four of seven daily activities are shown). Some interference was defined as a score of ≥3. There is a clear correlation between increased pain intensity and greater interference with daily activities and the enjoyment of life. Approximately 20%-30% of those with moderate pain (score 4) reported that the pain affected their daily activities. At least 51% of those who experienced high levels of pain (score 9-10) reported that pain interfered with each activity. Adapted with permission from Lydick *et al. *[31].

The substantial impact of acute-phase pain on QoL is being increasingly recognized, with QoL studies showing effects on patients' lives across all four health domains (Table 1) [18,19,24,26,36-38]. Many patients report that pain during the acute phase of HZ impacts 'quite a bit' or 'extremely' on their physical, role and social functioning [18]. Depression is also reported by patients with acute symptoms of HZ [18]. In a study of 50 patients with acute-phase HZ aged 54-94 years, a clear correlation between increased pain intensity and greater interference with activities (including general activity, work, sleep and enjoyment of life) was observed (Figure 1) [36]. At a moderate pain level of 4, 20%-30% reported interference but, at a high pain level of 9 or 10, ≥ 66% reported an effect on each activity.

**Table 1:** Impact of herpes zoster and post-herpetic neuralgia on all four health domains [18,23,24]

Physical	Psychological
Fatigue	Depression
Anorexia	Anxiety
Weight loss	Emotional distress
Reduced mobility	Difficulty concentrating
Physical inactivity	Fear
Insomnia	

**Social**	**Functional**

Withdrawal	Dressing, bathing, eating, mobility
Isolation	Travelling, cooking, housework, shopping
Attendance at fewer social gatherings	
Loss of independence	
Change in social role	

Different components of acute-phase pain impact on different health domains. This has been demonstrated by multivariate analyses, controlling for patients' demographic and clinical variables [18]. Data showed that significant independent contributions were made by sensory components of pain to poor physical functioning, affective components of pain to depression and psychological impairment and overall pain burden (sensory and affective combined) to poor social and role functioning.

Some elderly individuals may experience a permanent loss of independence, never fully regaining their lifestyles, interests and levels of activity after an acute episode of HZ [24].

#### Clinical features of PHN

PHN is characterized by constant and/or intermittent paroxysmal pain persisting for ≥90 days after the onset of HZ rash [8,10]. For some patients, PHN is a continuation of the painful symptoms of the acute phase of HZ; others experience completely different pains and sensations. Allodynia is present in ≥70% of patients and is usually considered to be the most distressing and debilitating PHN component [10,28]. Numbness and tingling also contribute to the PHN burden [10]. Clinically, there is an area of scarred skin, which denotes sensory loss and the wider area of dynamic mechanical allodynia. Other neuropathic features include burning, electric shocks, pins and needles and itching. The 'neuropathic itch' in PHN patients may lead to injury in those who scratch itchy skin so much that loses its protective sensation [39].

Most patients categorise the intensity of PHN as moderate-to-severe (pain scores ≥4 on a scale of 0-10) despite receiving analgesic agents [40]. The persistency of PHN means that patients have few periods of respite from erratic, painful and prolonged attacks.

#### Impact of PHN on Qol

**Figure 2:**
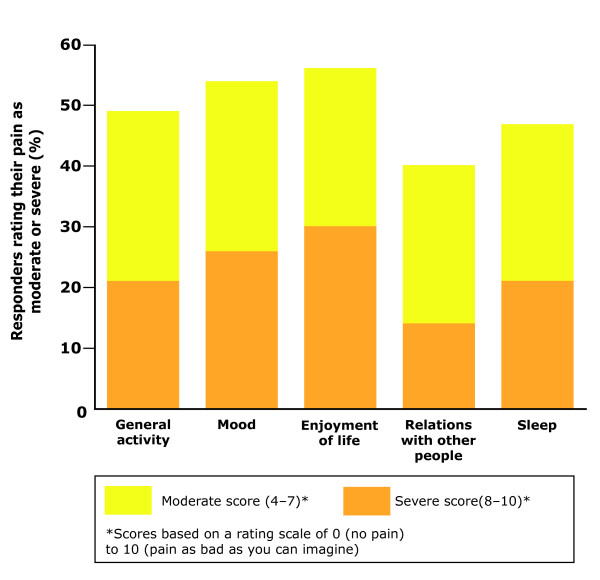
**Impact of post-herpetic neuralgia (PHN) across different aspects of quality-of-life (QoL) **[13]. A study assessed the impact of pain, medication use and QoL in 385 patients with PHN aged >65 years. Pain causes disruption across many aspects of life for PHN patients. As many as 40% of respondents said that pain moderately or severely affected their ability to carry out general activities. Forty-eight percent of patients commented that pain interfered moderately or severely with their enjoyment of life. Adapted with permission from Oster *et al. *[13].

Reduced QoL is a particular problem in patients whose pain persists as PHN. The impact of PHN on patients' lives is substantial and apparent across all domains of QoL (Table 1, Figure 2) [13,19,37,38,40-42]. There is a positive correlation between increasing pain severity and the extent of the negative impact on QoL [13]. In a study that assessed the impact of pain, medication use and QoL in 385 patients with PHN aged >65 years, 40% of respondents said that pain moderately to severely affected their ability to carry out general activities (Figure 2) [13]. In addition, 48% of patients commented that pain interfered moderately or severely with their enjoyment of life (Figure 2) [13].

PHN causes a loss of physical function, with patients experiencing fatigue, anorexia, weight loss, reduced mobility, physical inactivity, sleep disturbance (especially insomnia) and reductions in overall health [8,13]. PHN makes undertaking basic tasks (for example, bathing, dressing, eating) and complex activities (for example, travelling, performing household chores, shopping) difficult [8]. Institutionalization and a loss of autonomy can occur in elderly patients with PHN [24]. The loss of social contact, withdrawal and isolation can occur in patients with PHN because they experience reduced independence and participate less in social gatherings [8,24].

PHN may also affect patients' psychological wellbeing [13,34]. Psychosocial scores improve in patients who fully recover from the acute symptoms of HZ, but they remain low in patients who develop PHN [34]. Patients with intense pain are at greater risk of anxiety and depression than those who report milder pain [8,13]. Patients with PHN report difficulty in concentrating [24]. They also fear recurrences of PHN symptoms and may experience changes in their emotional roles within key relationships. A substantial proportion of patients receive medication for depression, anxiety and sleep disturbances related to PHN [40]. Some patients became suicidal as a direct consequence of PHN [38].

### Non-pain symptoms and complications of HZ may also affect QoL

Several typical symptoms of HZ can be debilitating for patients, even among those who do not experience clinically meaningful pain [43]. The main characteristic of the acute phase of HZ is the rash which, together with subsequent scarring, may be unsightly [28].

HZ can also give rise to non-pain complications, many of which can increase the risk of permanent or long-standing physical impairment [20]. Complications can be ophthalmic (for example, herpes zoster ophthalmicus), neurological (for example, cranial and peripheral nerve palsies), dermatological (for example, bacterial super-infection) or visceral (for example, pneumonia; Table 2) [20]. A degree of motor deficit is common in patients with HZ; severe and long-lasting paresis may rarely occur if HZ affects the cervical and lumbosacral dermatomes [44]. HZ patients may also be at greater risk of stroke. Two recent retrospective population-based studies in Taiwan found that HZ and herpes zoster ophthalmicus patients had a 1.31-fold and 4.52-fold higher risk of stroke, respectively, in the following year [45,46]. Almost one in 10 immunocompetent patients with HZ is affected by at least one non-pain complication [47]. The incidence of complications becomes more common with increasing age: patients aged >65 years have four-times the number of complications than do individuals younger than 35 years [48].

**Table 2:** The four main domains of complications (excluding post-herpetic neuralgia) identified in patients with acute herpes zoster (HZ)

Domain	Complications
Neurological [20]	Vertigo
	Cranial nerve palsies (for example, facial paresis)
	Hearing loss
	Varicella zoster virus encephalitis
	Motor neuropathy
	Myelitis
	Small-vessel encephalitis
	Granulomatous arteritis with secondary stroke

Ophthalmic [74]	Ptosis
	Scleritis
	Iridocyclitis
	Secondary glaucoma
	Cataract
	Keratitis
	Blindness
	Chorioretinitis

Dermatological [20]	Disseminated HZ
	Post-herpetic (persisting) pruritus
	Secondary bacterial skin infections (with subsequent scarring, cellulitis, septicaemia)

Visceral [20]	Pneumonia
	Peri-myocarditis
	Hepatitis
	Oesophagitis
	Myositis
	Arthritis

Complications associated with HZ and PHN place a heavy burden on individuals, who may suffer a permanent reduction in QoL even if the original HZ symptoms fully resolve [24].

### Wider impact of HZ and PHN on QoL

A survey by Weinke *et al. *was designed to capture the full course of HZ episodes from rash onset to pain resolution. The findings demonstrate the major and wide-ranging impact that HZ, and in particular PHN, have on patients' QoL [49]. Eleven thousand interviews were conducted in Germany to evaluate patient-rated pain in historic HZ episodes and interference with ADL. Screening questions identified 280 patients ≥50 years old with painful HZ diagnosed during the previous 5 years, of whom 32 patients developed PHN. Patients with PHN scored significantly worse across all outcomes related to pain and QoL than those with HZ [49]. The mean interference of pain on different aspects of QoL was highest for sleep, mood and work [49]. Three-quarters of patients experienced problems in carrying out daily activities, including work, studies, housework, family and leisure activities [49]. Approximately 60% of employed interviewees had to stop work at some point during the active disease period [49].

When patients' partners were asked how HZ or PHN had affected patients' QoL, most replied that the symptoms interfered considerably with patients' ADL. This was particularly evident for people who developed PHN [50]. Not only patients are affected by this disease: caring for patients with chronic diseases places a burden on their partners' personal lives and finances [51]. Patients' partners and family members may need to fill additional roles. Caring for a person with HZ or PHN may cause family members to take time off work [52]. Patients and their partners are less likely to attend or participate in social occasions [24,51].

HZ-related hospitalizations may contribute to the burden of HZ and PHN on patients and their families [53]. It is estimated that, in Europe, approximately 12,000 hospitalizations each year are due to HZ and its complications [38,48,54-56]. HZ complications which are likely to lead to hospitalization are PHN, encephalitis or meningitis and ocular complications [57]. Of 88,650 estimated annual cases of HZ in individuals ≥60 years old in England and Wales, an estimated 1750 (2%) hospitalizations are due primarily to HZ [53]. In Connecticut, USA, the mean annual hospitalization rate due to principal or secondary diagnosis of HZ over a 10-year period is 16.1/100,000 (range, 14.5-18.2) [58]. Almost 67% of these patients were aged >64 years.

### Comparisons between HZ or PHN and chronic conditions

The impact of acute HZ disease on patients' QoL is considerable [36], equalling that seen with common debilitating chronic conditions such as congestive heart failure, diabetes mellitus, myocardial infarction and clinical depression [59] (Table 3). HZ patients have an extremely low score for role limitations due to physical problems (scoring 19 out of 100, Table 3). They cannot accomplish daily duties or fulfil their roles as well as they would normally expect because of the acute effects of HZ. In patients with PHN, pain ranks very high compared with pain from other chronic conditions such as atypical facial pain, osteoarthritis and rheumatoid arthritis [30].

**Table 3:** Quality-of-life (QoL) scores obtained from patients with herpes zoster (HZ) [36] compared with different chronic diseases [59]

SF-36 domain	Within two weeks of HZ onset (*n *= 46)	Hyper-tension (*n *= 2089)	Congestive heart failure (*n *= 216)	Diabetes mellitus (*n *= 541)	Myocardial infarction (*n *= 107)	Depression (*n *= 502)
General health	71	63	**47**	56	59	53

Vitality	**45**	58	**44**	56	58	**40**

Body pain	**35**	72	63	69	73	59

Mental health	67	78	75	77	76	**46**

Role limitations due to emotional problems	**48**	77	64	76	73	**39**

Physical functioning	63	73	**48**	68	69	72

Role limitations due to physical problems	**19**	62	**34**	57	51	**44**

Social functioning	**52**	87	71	82	85	57

Modified from [31] and [59].

All scores were calculated using Short Form (SF)-36, a well-validated tool for measuring QoL that has a maximum score of 100; scores under 50 (in bold) represent low QoL

**Figure 3:**
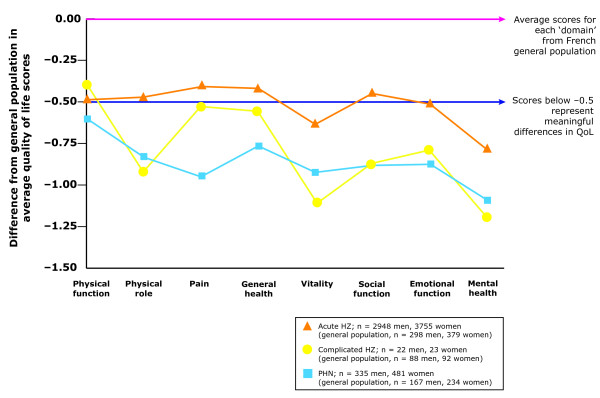
**Quality-of-life (QoL) scores in a French general population cohort versus patients with herpes zoster (HZ) or post-herpetic neuralgia (PHN) (Leplège, unpublished data; adapted from Figure 2 in Chidiac 2001 **[38]). The French general population cohort comprised healthy individuals and individuals with various chronic diseases (*n *= 3656). Average Short-Form-36 (SF-36) scores (eight domains) were calculated for the general French population and for patients with acute HZ, HZ-related complications, and PHN. The differences in scores between each patient group and the general French population are presented. Patients with HZ and PHN have poorer QoL scores than the general-population group. Clinically meaningful differences (scores below -0.5) are observed for some domains in patients with HZ, and for all domains in patients with complicated HZ or PHN. All three groups of patients scored low for the SF-36 domains of vitality and mental health.

QoL analysis has been undertaken using data from a general-population group (including healthy people and those with a wide range of chronic conditions such as arthritis, chronic lung disease, congestive heart failure, diabetes, myocardial infarction or angina and hypertension; Leplège, unpublished data). Compared with the mean QoL level for each domain in the general population, patients suffering from HZ have poorer QoL scores (Figure 3) [38]. During the acute phase of HZ, the impact is clinically meaningful for some domains. For patients suffering from complications or PHN, the impact is higher and all the domains are affected (Figure 3) [38].

### Current challenges in the treatment of HZ and PHN

Treatment of patients with HZ aims to accelerate rash healing, relieve pain and reduce complications [5]. Current treatments are effective in some patients, but therapies may show limited efficacy and poor tolerability in others (particularly in the elderly; Table 4).

**Table 4:** Effect on pain and quality-of-life of agents used to treat herpes zoster (HZ) and/or post-herpetic neuralgia (PHN)

Treatment	Advantages	Disadvantages
Antiviral agents	Relieve acute HZ pain and accelerate lesion healing if administered within 72 h of acute-symptom onset [8]. Few adverse effects [75,76]. May slightly reduce PHN symptoms and their duration [5,8,60,67,71,77-79].	Administration within 72 h is usually not achievable [8]. In clinical trials, 20%-30% of treated patients still develop PHN [37,71].

Corticosteroids	Reduce intensity of pain and overall duration of the acute phase [37,80,81]. Significantly accelerate time to uninterrupted sleep, return to daily activity, and cessation of analgesic therapy.	Do not prevent PHN and produce significant adverse events in older adults; their routine use is therefore not recommended in elderly patients with HZ [37].

Simple analgesics	May reduce pain in HZ and PHN [11,75].	Few trials assessing efficacy in HZ or PHN.

Tricyclic antidepressants	Provide effective pain relief in PHN patients (numbers needed to treat = 2.8) and may possibly provide benefits through sedative actions given that PHN can induce sleep disturbances and anxiety [67].	Side-effects may cause further QoL problems. Patients do not regain the level of life-satisfaction that they had before PHN developed [43,67].

Antiepileptics	Gabapentin and pregabalin offer reasonable relief for PHN [82-84].	Levels of pain relief are not associated with similar improvements in QoL scores [82-84].

Opioids	Maximum tolerable doses may reduce PHN pain [67].	Side-effects are common and troublesome, particularly for elderly patients; overall benefits are therefore limited [67].

Topical agents	Lidocaine patch provides some pain relief and has few side-effects [85]. Capsaicin dermal patch significantly reduces pain [86].	Discomfort experienced with capsaicin formulations; overall benefits are therefore limited [11]. Pain relief with capsaicin dermal patch is not associated with improved QoL in PHN [86].

Epidural therapies and nerve blocks	Continuous epidural local anaesthetic has been shown to effectively treat acute-phase HZ pain [87]. Prolonged (not single-dose or short-term) epidural local anaesthetic blockade with corticosteroid may provide some protection against PHN [88].	Single-dose epidural local anaesthetic/steroid does not prevent PHN [89]. Epidural corticosteroid has potential risk, and anecdotal evidence has not supported the benefit. Prolonged epidural local anaesthetic blockade is not practical for widespread use and carries some risk [88].

Despite the development of treatment guidelines by the International Herpes Management Forum [60], HZ management is complex [61]. The guidelines recommend oral antiviral agents for 7 days in patients with HZ who are at risk of developing PHN (patients aged >50 years with severe acute pain, severe rash, or significant prodromal symptoms) [60]. Studies have shown that aciclovir, valaciclovir and famciclovir decrease viral shedding, relieve acute phase pain, reduce the formation of new lesions and accelerate healing [5,62,63]. In general, these treatments are well tolerated, even by older patients [37]. By reducing viral replication, they may decrease the duration of acute-phase pain and PHN [4,5].

To be effective, antiviral agents must be administered within 72 h of acute-symptom onset [60], which may be difficult because of delays in patients seeking medical advice and delays in reaching diagnosis (for example, due to presentation of unusual symptoms) [8]. Often, viral activity and neuronal damage have been ongoing for several days before appropriate treatment is given [4]. Even with prompt treatment, antiviral treatment does not prevent all cases of PHN [37,64]. A recent Cochrane Review demonstrated that oral acyclovir does not significantly reduce PHN incidence [64]. The Cochrane review highlighted that there is insufficient evidence from randomized controlled trials to determine if other antiviral agents prevent PHN and if these treatments have a beneficial impact on QoL [64]. Twenty studies of antiviral treatment for acute HZ were identified, but 14 were excluded from the Cochrane review, mainly because of limitations with the study design.

**Figure 4:**
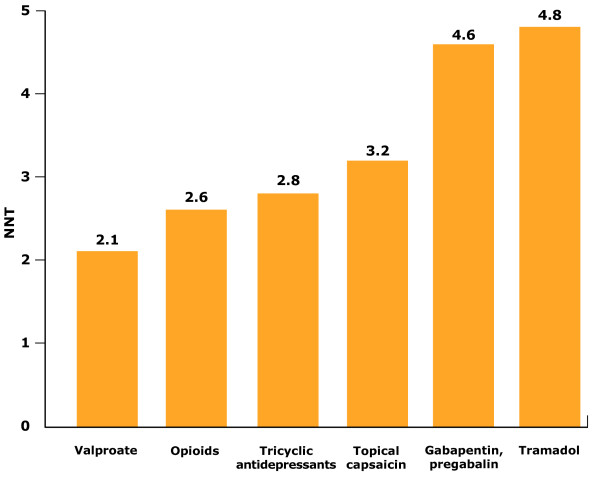
**Number needed to treat with common pain therapies to obtain 50% pain relief in one patient **[68]. Data highlight the limited relief from pain obtained in patients with post-herpetic neuralgia (PHN). For some treatments, approximately four patients with PHN need to be treated to achieve 50% pain relief in one patient.

A recent Canadian study of patients with HZ rash or pain showed that pain resolution is associated with recovery of QoL to a pre-morbid state (personal communication). Data showed that the median duration of pain was equal to the median duration of QoL impairment. Once established, the pain associated with HZ or PHN is difficult to treat [65]. PHN remains largely refractory to pharmacological treatments and prevention strategies [66]. Data show that >50% of patients require more than one prescription drug for PHN [13]. Despite treatment, symptomatic relief is obtained in only 50% of patients [67]. Patients with PHN experience significantly less pain relief than those with HZ, despite receiving more pain medications (mean, 2.2 versus 1.6) [49]. Studies have assessed the 'number needed to treat' for existing PHN therapies (how many patients need to be treated n order to achieve at least 50% pain relief in one patient compared with the controls). Data highlight the limited pain relief obtained in PHN patients. For some standard treatments, approximately four patients with PHN need to be treated in order to achieve pain relief in one patient (Figure 4) [68].

As patients with PHN may find it difficult to tolerate pain therapies, drug doses are often titrated. This may result in several weeks of suboptimal treatment, during which it is unclear if (or when) the drugs will become effective [67]. Therapeutic compliance is also difficult to achieve. Almost 50% of patients with PHN do not discuss their symptoms regularly with physicians [13]. One in 10 is troubled substantially by the side-effects of PHN treatments [13,24].

Treatment dissatisfaction is high in patients with PHN. In one study, only 14% of patients aged >65 years were satisfied with pain medication for PHN [13]. In the survey by Weinke *et al*. [49], patient-reported satisfaction with HZ treatments was significantly lower in patients with PHN versus those with HZ (6.8 versus 8.3; scale from 0, 'not satisfied' to 10, 'very satisfied').

### Vaccination may reduce the burden of HZ disease

Current treatment strategies for HZ and PHN are only partially effective, so reducing the burden of HZ disease remains an area of considerable need [11]. A solution is for proactive strategies to focus on HZ prevention [67]. At least one study has shown that, compared with people who have not experienced HZ, those who have had HZ or PHN place higher value on preventing this disease. In this study, both groups questioned (community members and patients with HZ or PHN) said that they would be willing to pay substantial amounts of money to prevent HZ [69].

**Figure 5:**
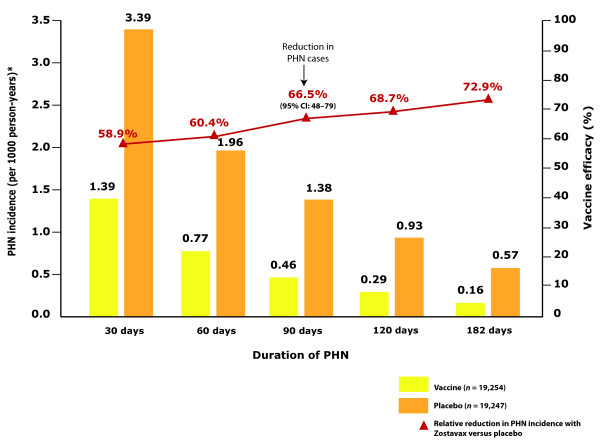
**Duration of pain in vaccine recipients in the Shingles Prevention Study **[70]. The graph compares vaccine recipients who developed herpes zoster and post-herpetic neuralgia (PHN; 315/19,254 recipients) with placebo recipients who developed disease (641/19,247 recipients). Compared with placebo, Zostavax reduced PHN incidence defined as pain at different cut-off times for the duration of pain. Pain persisting at 90 days was reduced by 67%. * For the total population and the sub-groups stratified according to sex, the incidence of PHN in each treatment group (vaccine or placebo) was the weighted average of the observed incidence of PHN stratified according to age group, with weights proportional to the total number of person-years of follow-up in each age group.

The clinical rationale for the development of an HZ vaccine has centred on reducing the severity of HZ and PHN. Studies have shown that the live attenuated Oka/Merck VZV vaccine ('zoster vaccine') also reduced the incidence of HZ and PHN in addition to burden of illness (a severity-by-duration measure of the total pain and discomfort associated with a disease). Evidence that the zoster vaccine reduces HZ burden in an ageing population is derived from the Shingles Prevention Study [70]. The Shingles Prevention Study had a randomized, double-blind, placebo-controlled design and assessed HZ burden of illness and PHN incidence (pain rated as ≥3 on a 0-10 scale ≥90 days after rash onset) in >38,000 people aged ≥60 years who received zoster vaccine or placebo. Compared with placebo, vaccination significantly reduced HZ burden of illness by 61.1%. Vaccine recipients had a significantly reduced incidence of HZ (-51%) and PHN (-67%) compared with placebo [70,71]. HZ symptoms in the vaccine group were, in general, milder and of shorter duration than those in the placebo group [70,71]. Vaccination reduced PHN incidence defined using alternative cut-off times for the duration (persistence) of pain; the reduction in incidence was 72.9% at 180 days (Figure 5) [70,71]. Although the reduction in HZ incidence with vaccine compared with placebo was less apparent in patients who were aged ≥70 years versus those aged 60-69 years, the reductions in HZ burden of illness and PHN were preserved in the older population [70].

In an adverse event substudy of the Shingles Prevention Study, rates of serious adverse events were higher in the vaccine group (1.9%) than in the placebo group (1.3%) [70]. Injection site reactions were more frequent among vaccine recipients than those taking placebo (48.3% versus 16.6%, respectively), but were generally mild.

The impact of vaccination on QoL was assessed in the entire study population of the Shingles Prevention Study using ZBPI and ZIQ questionnaires. Mean ZBPI and ZIQ scores, which rated the 'interference' of HZ and PHN with ADL, were used to calculate a 'severity of interference score'. For the entire study population, zoster vaccination reduced the ZBPI severity of interference by 66% (95% confidence interval; CI: 55, 74) and the ZIQ severity of interference by 68% (95% CI: 57, 77). In individuals who developed HZ, vaccination reduced the ZBPI severity of interference by 31% (95% CI: 12, 51) and the ZIQ severity of interference by 35% (95% CI: 13, 57). Zoster vaccination therefore reduced the burden of HZ-related interference with ADL by about two-thirds in a population of older adults and by about one-third in vaccine recipients who developed disease [72]. Much of the effectiveness of the zoster vaccination was due to the prevention of HZ episodes [70-73].

## Summary

HZ is a common disease that can substantially reduce patients' QoL and functional status. HZ is rare in young individuals, but there is a marked increase in risk after 50 years of age. The consequences of HZ are a particular problem in older individuals. In part, this may be due to slow healing of nerve damage and inflammatory scarring in an ageing nervous system. The number of cases of HZ and PHN is expected to increase in coming decades due to the steady rise in the mean age of the population.

Pain is the major symptom that affects the QoL and ADL of patients and is usually present across all phases of HZ disease. In patients whose pain persists for months or years as PHN, the effects on patients' lives can be devastating. The negative effects of HZ and PHN on physical, social, functional and psychological health are as substantial as the effects described by patients with other chronic health conditions. Non-pain-related complications of HZ may also contribute to a reduced QoL. HZ and PHN can also have significant consequences for patients' partners, relatives and social circle.

To be effective, antiviral medication needs to be started within 72 h of the onset of acute symptoms. Antiviral agents show limited efficacy if administered within 72 h of acute-symptom onset. Treatment within this time is often not achievable and patients may present with pain that is established and difficult to treat. Current treatments for PHN are largely suboptimal and often accompanied by intolerable side-effects. Awareness of these challenges and the substantial burden of HZ and PHN on patients' QoL may lead to improved prevention and management strategies.

Zoster vaccine has been demonstrated to attenuate the severity of HZ disease and significantly reduce the incidence of HZ and PHN. The introduction of a prophylactic vaccination scheme may achieve a significant positive impact on the incidence and course of HZ disease, thereby improving patients' QoL.

## Abbreviations

ADL: activities of daily living; CI: confidence interval; HZ: herpes zoster; PHN: post-herpetic neuralgia; QoL: quality-of-life; VZV: varicella-zoster virus; ZBPI: Zoster Brief Pain Inventory; ZIQ: Zoster Impact Questionnaire.

## Competing interests

RWJ received honoraria as consultant and/or lecturer for Merck, Sanofi Pasteur MSD, Novartis, Astellas, GSK and Merck Frosst. DB received support from Sanofi Pasteur MSD, Sanofi Aventis, Pfizer, Boehringer Ingelheim, Eli Lilly. GK received an honoraria and support to attend scientific meetings from Sanofi Pasteur MSD, GSK, Novartis, AstraZeneca, Sanofi Aventis, Pfizer and Roche. AL was a consultant for Sanofi Pasteur MSD. KES received a grant support from Merck and Wyeth and was a consultant for Merck and GSK. TW received honoraria as a consultant and/or lecturer for GSK, Novartis Vaccines and Sanofi Pasteur MSD.

## Authors' contributions

All authors contributed to the content of this manuscript and have read and approved the final draft.

## Pre-publication history

The pre-publication history for this paper can be accessed here:

http://www.biomedcentral.com/1741-7015/8/37/prepub
